# Treatment Outcomes of Pelvic Organ Prolapse Seven Years After the Introduction of Sacrocolpopexy at a Single Institution

**DOI:** 10.7759/cureus.89647

**Published:** 2025-08-08

**Authors:** Yuka Oi, Tatuya Matsunaga, Sayako Nakagawa, Mihoko Dofutsu, Keiko Segawa, Mai Shimura, Yoshiko Arata, Atsuko Furuno, Kayo Katayama, Osamu Chaki

**Affiliations:** 1 Obstetrics and Gynecology, Yokohama Rosai Hospital, Yokohama, JPN; 2 Obstetrics and Gynecology, Saiseikai Yokohamashi Nanbu Hospital, Yokohama, JPN

**Keywords:** laparoscopic sacrocolpopexy, native tissue repair, pelvic organ prolapse, robotic sacrocolpopexy, sacrocolpopexy

## Abstract

Introduction

Pelvic organ prolapse (POP) affects up to 30% of women during their lifetime and significantly impairs quality of life. In Japan, laparoscopic sacrocolpopexy was covered by national insurance starting in 2014 and has become an established treatment option.

Objective

This study evaluates the long-term outcomes of POP surgery, including recurrence and complications, seven years after the introduction of sacrocolpopexy at our institution.

Methods

Our institution introduced sacrocolpopexy in 2016, while continuing to perform conventional vaginal surgery. Sacrocolpopexy was primarily offered to patients under 80 years of age without diabetes; for other patients, vaginal surgery was generally chosen. Among vaginal surgeries, patients with a favorable general condition underwent native tissue repair (NTR). We retrospectively reviewed the medical records of patients who underwent sacrocolpopexy or NTR between April 2016 and June 2023.

Results

During the period, 90 patients underwent POP surgery, with 37 undergoing sacrocolpopexy and 38 receiving NTR; these 75 cases were included in the analysis. In the sacrocolpopexy group, the median time from symptom onset to surgery was 24 months, and 22 patients had used a pessary preoperatively. The median operative time for sacrocolpopexy was 196 minutes, with minimal bleeding and no intraoperative complications. Postoperative adverse events after sacrocolpopexy included one case each of delirium and acute cholecystitis. Recurrence of stage II or higher POP was observed in three patients in the sacrocolpopexy group; two of these had preoperative stage IV disease and had waited 60 months from symptom onset to surgery. In the NTR group, the median time to surgery was 42 months, and 32 patients had used a pessary preoperatively. The median operative time for NTR was 89 minutes, with minimal bleeding and no intraoperative complications. One postoperative complication (vaginal cuff hematoma) was observed after NTR. Recurrence of POP stage II or higher occurred in three NTR patients, all of whom were stage III or higher preoperatively.

Conclusion

Sacrocolpopexy and NTR are effective surgical options for POP, particularly in patients who do not respond to conservative management. Although sacrocolpopexy and NTR had a low complication and recurrence rate, it is important to bear in mind that recurrence is possible.

## Introduction

Pelvic organ prolapse (POP) negatively affects the quality of life of up to 30% of women [1] and 40% to 60% of parous women [2]. As many as 11.1% of women may undergo surgery for POP between 20 and 80 years of age [3]. Although the prevalence of POP in Japan is unclear, African American women reportedly have a lower POP incidence, Hispanic women are more prone to uterine prolapse, and Asian women are more likely to develop cystocele [4].

POP occurs when the pelvic support tissues fail due to factors such as pregnancy and childbirth, obesity, and genetic musculoskeletal abnormalities. Symptoms include vaginal bulging, pain, bleeding, urinary disturbances, and sexual dysfunction, and various treatment strategies have been proposed. Treatment options are broadly classified into conservative and surgical approaches. Conservative therapies include pelvic floor muscle exercises and pessaries. Surgical approaches are categorized into vaginal and abdominal procedures. Vaginal procedures include native tissue repair (NTR), colpocleisis, and transvaginal mesh surgery. Abdominal procedures include sacrocolpopexy and laparoscopic uterosacral ligament suspension (L-USLS). Abdominal sacrocolpopexy was first reported by Lane in 1962 and is characterized by a high cure rate, although it initially required laparotomy [5]. In 1994, Nezhat et al. [6] reported laparoscopic sacrocolpopexy (LSC). In Japan, LSC has been covered by insurance since 2014 [7], followed by robotic-assisted sacrocolpopexy (RSC) in 2020 [8].

With insurance coverage, our hospital introduced LSC in 2016, but we have continued to perform vaginal surgeries, especially NTR, which was standard procedure. We also introduced RSC in 2021. Seven years have passed since the introduction of sacrocolpopexy, and the long-term prognosis, which is important in POP surgery, has become clear.

This study evaluates the efficacy and safety of sacrocolpopexy and NTR for POP at our institution. Efficacy was defined as the recurrence rate after surgical treatment, and safety was defined as intraoperative complications and postoperative adverse events.

The abstract of this paper was presented at the 38th Japan Society for Menopause and Women's Health congress on December 2, 2023.

## Materials and methods

This retrospective review included POP surgeries performed at our hospital from April 2016 to June 2023. The following data were extracted from medical records: age, body mass index, history of pregnancy and delivery, time from onset to surgery, pessary treatment before surgery (yes/no) and its duration, degree of POP at the time of surgery, operative time, blood loss, intraoperative complications, postoperative adverse events (during hospitalization and after discharge), recurrence (yes/no), degree of POP at recurrence, and timing of recurrence.

The degree of POP was quantitatively assessed using the POP quantitative description system (hereafter referred to as POP-Q) stage classification at the time of the outpatient visit. Intraoperative complications were defined as adverse events related to the surgery, and postoperative adverse events, including those not directly related to the surgery, were evaluated using the Clavien-Dindo classification. Recurrence was defined as POP-Q stage II or higher.

Patients were eligible for surgery if they were POP-Q stage II or higher and desired surgery. If patients did not desire surgery, pessary therapy was selected. Patients were selected according to the ECOG performance-status score (PS) and De Lancy site of injury. For De Lancy 1 and De Lancy 2 disorders, colpocleisis was performed if the PS was less than or equal to 1. Sacrocolpopexy was selected if the PS was 0, the patient was younger than 80 years, had no diabetes mellitus, and had no other contraindications such as immunodeficiency or vertebral degeneration. Otherwise, vaginal total hysterectomy + uterosacral colpopexy + anterior and posterior colporrhaphy were performed as the NTR method. In the case of a myoma or ovarian cyst that required transabdominal surgery but was not amenable to sacrocolpopexy, L-USLS was performed after laparoscopic surgery for the relevant lesion.

Sacrocolpopexy was performed as described below. The uterine body was removed while the cervix was left intact. Polypropylene or polytetrafluoroethylene mesh was used to suture the anterior vaginal wall with a nonabsorbable suture. The mesh was sutured to the Cape angle (anterior longitudinal ligament in front of the sacrum). In the double mesh technique, additionally, the anal eminence was exposed, and a mesh was sutured to the anal eminence, the posterior vaginal wall, and the posterior wall of the cervix. Concomitant vaginal surgery was not performed in any of the patients.

Stata13.1™ (Stata Corp., College Station, TX, USA) was used for analysis. Categorical variables were compared using the χ-square test, and continuous variables were compared using the Mann-Whitney U test, with p <0.05 deemed to be significant. Considering the number of cases, Fisher's exact test was performed instead of multivariate analysis, with p <0.05 deemed to be significant.

## Results

From April 2016 to June 2023, 90 POP surgeries were performed, of which 37 were sacrocolpopexy (26 LSC and 11 RSC) procedures and 38 were NTRs; these 75 cases were included in the analysis.

**Figure 1:**
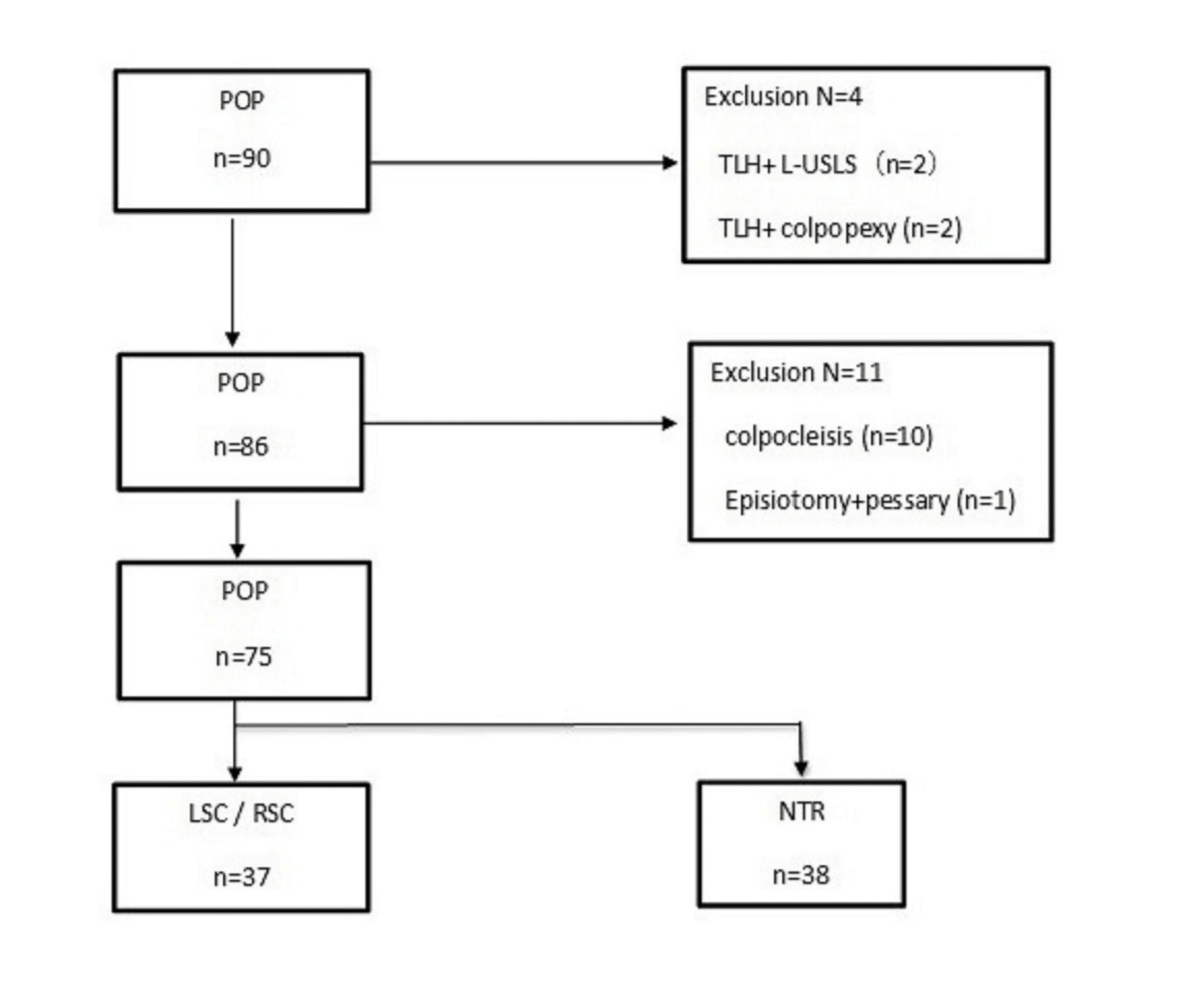
Flowchart showing the flow of patients undergoing pelvic organ prolapse surgery at our institution LSC, laparoscopic sacrocolpopexy; L-USLS, laparoscopic uterosacral ligament suspension; NTR, native tissue repair; POP, pelvic organ prolapse; RSC, robotic sacrocolpopexy; TLH, total laparoscopic hysterectomy

The flowchart for participant selection is shown in Figure 1, and the patient characteristics are shown in Table 1.

**Table 1:** Baseline characteristics of patients who underwent pelvic organ prolapse surgery at our institution Categorical variables were compared using the χ-square test, and continuous variables were compared using the Mann-Whitney U test, with p <0.05 deemed to be significant. LSC, laparoscopic sacrocolpopexy; POP-Q, pelvic organ prolapse quantitative description system; RSC, robotic sacrocolpopexy; NTR, native tissue repair

Characteristic	LSC/RSC	NTR	p-value
Number of patients	37	38	-
Age, years (mean±SD)	68.2±8.4	73.2±6.9	0.013
Body mass index, kg/m^2 ^(mean±SD)	23.5±3.4	23.4±3.2	0.814
Parity, median (IQR)	2 (2–3)	2 (2–3)	0.455
POP-Q, no. (%)			
II	7 (18.9)	9 (23.6)	0.779
III	18 (48.6)	12 (31.6)	0.161
IV	12 (32.4)	17 (44.7)	0.345
ECOG performance status score, no. (%)			
0	37 (100)	38 (100)	-
1	0	0	-
Preoperative pessary insertion (yes), no. (%)	22 (59.5)	32 (84.2)	0.017
Duration of pessary usage, months (median [range])	3 (0–120)	1 (0–144)	0.113

The mean age for sacrocolpopexy was 68.2 years, younger than that for NTR (73.2 years). Preoperative use of pessaries was 22 (59.5%) for sacrocolpopexy and 32 (84.2%) for NTR. Some patients had been using pessaries for more than 10 years. The median time from symptom awareness to surgery was 24 months for sacrocolpopexy and 42 months for NTR, and in some cases, it was more than 10 years. Table 2 shows the perioperative results; LSC was used before 2022 and RSC after 2022.

**Table 2:** Perioperative outcomes of patients who underwent pelvic organ prolapse surgery at our institution Categorical variables were compared using the χ-square test, and continuous variables were compared using the Mann-Whitney U test, with p <0.05 deemed to be significant. LSC, laparoscopic sacrocolpopexy; RSC, robotic sacrocolpopexy; NTR, native tissue repair

Perioperative outcomes	LSC/RSC	NTR	p-value
Number of patients	37	38	-
Duration between the onset of symptoms and surgery, months (median [range])	24 (3–144)	42 (2–240)	0.347
Operative time, minutes (median [IQR])	196 (173–235)	89 (79–108)	<0.001
Blood loss, mL, median (IQR)	0 (0–0)	0 (0–37)	<0.001
Duration of hospital stay after surgery, days, median (IQR)	4 (3–4)	4 (4–4)	0.0019
Intraoperative complications	0	0	-
Postoperative complications	2	1	0.615
Clavien-Dindo classification			
Grade 1	1	1	-
Grade 2	0	0	-
Grade 3	1	0	-
Grade 4	0	0	-
Grade 5	0	0	-
Follow-up duration, months, median (IQR)	48 (15–84)	6 (2–36)	<0.001
Subjective prolapse, number (%)	5 (13.5)	8 (21.1)	0.389
Objective prolapse, all compartments prolapse grade >1, number (%)	3 (8.1)	3 (7.9)	0.972
Time of prolapse after surgery, number of patients/number of patients followed-up			
6 months	0/13	1/28	-
12 months	2/3	1/4	-
24 months	0/4	0/5	-
36 months	0/8	1/1	-
48 months	0/3	0/0	-
60 months	0/4	0/0	-
72 months	1/2	0/0	-

Postoperative complications of sacrocolpopexy included one case of postoperative delirium and one case of acute cholecystitis. No mesh-related complications were observed.

One case of vaginal hematoma was noted as a postoperative complication of NTR. The operative time was shorter with NTR, and blood loss was less with sacrocolpopexy. Hospital stay was shorter for sacrocolpopexy. Postoperative observation was significantly longer for sacrocolpopexy. Recurrence rates did not differ significantly.

Three recurrent cases of POP-Q stage II or higher after sacrocolpopexy are shown in Table 3. The time from symptom onset to surgery was 24 months or longer. The two patients who experienced early recurrence underwent surgery 60 months after onset. In addition, both early recurrences were preoperative POP-Q stage IV. Fisher's exact test was used to compare POP-Q4 and less than 4. The result was a p-value of 0.56, and no statistical difference was demonstrated.

**Table 3:** Recurrence of pelvic organ prolapse after sacrocolpopexy LSC, laparoscopic sacrocolpopexy; POP-Q, pelvic organ prolapse quantitative description system

Characteristic	Patient 1	Patient 2	Patient 3
Age, years	64	72	55
Duration between the onset of symptoms and surgery, months	24	60	60
Preoperative POP-Q stage	3	4	4
Ba	4	3	3
C	0	4	5
Bp	-3	-2	3
Duration between surgery and recurrence of prolapse, months	60	8	7
Recurrence POP-Q stage	3	3	4
Ba	2	2	1
C	-5	-5	3
Bp	-3	-3	1
Treatment	None	Pessary	Surgery (re-LSC)
Duration of follow-up, months	79	77	50
Recurrence POP-Q stage	3	4	4
Ba	2	3	1
C	-5	2	2
Bp	-3	-1	-1
Treatment	Observation	Surgery (colpocleisis)	Observation

The first patient was 64 years old. Surgery was performed 24 months after symptom onset. Surgery was requested due to discomfort after one month of pessary use (prescribed by a nearby doctor). Postoperative examination at the 60th month revealed that the anterior vaginal wall had dropped to +3. She is considering colpocleisis after weight loss, but she does not wish to undergo further treatment at this time and continues to visit the clinic regularly.

The second patient was 72 years old. Surgery was performed 60 months after symptom onset. She had no history of hospital visits or pessary use. The anterior vaginal wall drooped to +2 at 12 months postoperatively, and colpocleisis was performed at 72 months after LSC. Since then, the patient has been under observation with no recurrence.

The third patient was 55 years old, and surgery was performed 60 months after symptom onset. The patient had no history of hospital visits and no history of pessary use. Nine months after surgery, the uterovaginal area dropped to +3, and LSC was performed again. The mesh was removed from the anterior vaginal wall and cervix but not from the cape angle. The mesh was reapplied to the cape angle, anterior vaginal wall, and cervix. Nine months after the reoperation, the uterovaginal area dropped to +2 again. She has no desire for further treatment at this time and continues to visit the clinic regularly.

In the NTR group, three patients experienced recurrent POP-Q stage II or higher (Table 4). The median time from onset to surgery in the NTR group (n = 23) was 42 months, but it was longer for two patients who experienced recurrent POP (patient 2 = 240 months and patient 3 = 48 months). These two patients underwent surgical treatment more than four years after symptom onset.

**Table 4:** Recurrence of pelvic organ prolapse after native tissue repair POP-Q, pelvic organ prolapse quantitative description system

Characteristic	Patient 1	Patient 2	Patient 3
Age, years	57	70	72
Duration between the onset of symptoms and surgery, months	36	240	48
Preoperative POP-Q stage	3	3	2
Ba	3	3	0
C	-1	2	0
Bp	-3	0	-1
Duration between surgery and recurrence of prolapse, months	36	7	3
Recurrence POP-Q stage	2	2	2
Ba	1	1	1
C	0	-2	-2
Bp	-3	0	0
Treatment	Observation	Observation	Observation
Duration of follow-up	12	12	7

In the NTR group, one patient experienced POP recurrence three years after surgery to treat a recurrent bladder mass, POP-Q stage II. In the second patient, a bladder mass POP-Q stage II and a rectal mass POP-Q stage II were detected seven months after surgery, but the patient did not wish to undergo further treatment, and the consultation ended 12 months after surgery. The third patient showed a recurrent rectal mass, POP-Q stage II, three months after surgery, but the patient did not desire further treatment and the consultation ended seven months after surgery.

## Discussion

Both sacrocolpopexy and NTR demonstrated low complication and recurrence rates in this study. However, patients with preoperative POP-Q stage IV were more likely to experience recurrence, irrespective of the surgical approach. Additionally, a longer interval between symptom onset and surgery appeared to contribute to recurrence.

Regarding surgical outcomes, the operative times for sacrocolpopexy and NTR at our institution are comparable to previously published reports [7-12], with minimal blood loss and few perioperative complications, indicating the safety of these procedures. The difference in the hospital stay duration between the two groups reflects our institutional discharge protocols, typically the third or fourth postoperative day for laparoscopic surgery and the fourth day for vaginal surgery, rather than differences in recovery. The longer follow-up period observed in the sacrocolpopexy group was due to lower follow-up compliance among NTR patients. The recurrence rate for NTR was 7.9% (3/38) in this study. However, it is plausible that extended follow-up in the NTR might reveal a higher recurrence rate.

The recurrence rate was 8.1% (3/37) for sacrocolpopexy, 11.5% (3/26) for LSC alone, excluding RSC, and 10.3% (3/29) for double mesh procedures, consistent with previous literature. Reported recurrence rates varied across studies: 3.4% at 18 months (Narushima et al. [7]); 8.0% at 12 months (Moriyama et al. [9]); 3.4%-11.5% at 20 months (Bacle et al. [10]); and 18.3% at 20 months (Costantini et al. [11]). Notably, Costantini et al. [11] reported that recurrence was more common in the anterior vaginal wall and often within one year. In our study, two recurrences following sacrocolpopexy occurred between 6 and 12 months postoperatively, and one occurred after five years. Some institutions perform bladder fundoplication at the same time as sacrocolpopexy to prevent recurrence in the anterior vaginal wall, although the usefulness of this protocol has not been confirmed. Hence, we do not perform simultaneous surgery at our institution. Additionally, current evidence does not clearly distinguish recurrence outcomes between LSC and RSC [13-14], and thus we did not compare LSC and RSC in this study. Future differences in recurrence rates between double and single mesh methods may emerge. Although our institution transitioned to single mesh to reduce mesh-related complications, this study was not designed to compare these methods. Given that a single mesh was used in only eight patients, with an average follow-up of 16 months, its impact on overall outcomes is likely limited. Further research is necessary.

Regarding efficacy, our findings support the efficacy of surgery for treating POP. Conservative treatments, such as pelvic floor muscle exercises, pessaries, and the FemiCushion™, are viable options, but their limitations are well documented. Pessary therapy, for example, has a surgical conversion rate of approximately 54% [15]. At our institution, many patients opted for surgery following prolonged conservative management, indicating a common treatment trajectory. While we did not compare conservative and surgical methods directly, surgical intervention remains an effective treatment option for appropriately selected patients.

Regarding recurrence of POP after surgery, previous studies reported higher recurrence rates among patients with preoperative POP-Q stage IV undergoing NTR or sacrocolpopexy [8,16,17]. In our study, two of the three patients with recurrence were preoperative POP-Q stage IV, a result consistent with existing reports.

All of the recurrences in our study occurred in patients who waited over 24 months from symptom onset to surgery; in two cases, the delay was approximately 60 months. This suggests that prolonged mechanical loading and possible inflammation may weaken pelvic support structures, highlighting the potential benefits of earlier surgical intervention. However, this observation should be interpreted with caution due to the small sample size, potential inaccuracies in symptom onset reporting, and the multifactorial nature of POP pathophysiology.

Both sacrocolpopexy and NTR achieved favorable outcomes and are appropriate surgical options for POP. The range of surgical options continues to expand, including transvaginal natural orifice transluminal endoscopic surgery (vNOTES), offering clinicians increased flexibility in tailoring treatment to individual patients and institutional capabilities.

This study has several limitations. This was a single-center, retrospective descriptive study with a limited number of patients. The low number of recurrence events makes it impossible to prove a correlation. In addition, we did not quantitatively assess quality of life pre-or postoperatively, and thus the patient-reported outcomes are inadequate. Additionally, shorter follow-up times in the NTR group may have underestimated recurrences. Nonetheless, long-term data on POP surgery in Japan remain scarce, and this study contributes valuable information to the field. Large-scale prospective studies are needed to evaluate quality of life, recurrence rates, and cost-effectiveness.

## Conclusions

Sacrocolpopexy and NTR are effective surgical options for POP, particularly in patients who have not responded to conservative management. Although sacrocolpopexy and NTR had a low complication and recurrence rate, it is important to bear in mind that recurrence is possible.
